# Gypsum (CaSO_4_·2H_2_O) Scaling on Polybenzimidazole and Cellulose Acetate Hollow Fiber Membranes under Forward Osmosis

**DOI:** 10.3390/membranes3040354

**Published:** 2013-11-08

**Authors:** Si Cong Chen, Jincai Su, Feng-Jiang Fu, Baoxia Mi, Tai-Shung Chung

**Affiliations:** 1 NUS Graduate School for Integrative Sciences & Engineering (NGS), National University of Singapore, 28 Medical Drive, 117456, Singapore; E-Mail: a0091934@nus.edu.sg; 2 Department of Chemical and Biomolecular Engineering, National University of Singapore, 4 Engineering Drive 4, 117576, Singapore; E-Mail: cheff@nus.edu.sg; 3 Mann+Hummel Ultra-Flo Pte Ltd., 18 Tuas Avenue 8, 639233, Singapore; E-Mail: jcsu2991@gmail.com; 4 Department of Civil and Environmental Engineering, University of Maryland, College Park, MD 20742, USA; E-Mail: bmi@umd.edu; 5 Water Desalination & Reuse (WDR) Center, King Abdullah University of Science and Technology, Thuwal 23955-6900, Saudi Arabia

**Keywords:** forward osmosis, fouling, gypsum scaling, polybenzimidazole, polyhedral oligomeric silsesquioxane, cellulose acetate

## Abstract

We have examined the gypsum (CaSO_4_·2H_2_O) scaling phenomena on membranes with different physicochemical properties in forward osmosis (FO) processes. Three hollow fiber membranes made of (1) cellulose acetate (CA), (2) polybenzimidazole (PBI)/polyethersulfone (PES) and (3) PBI-polyhedral oligomeric silsesquioxane (POSS)/polyacrylonitrile (PAN) were studied. For the first time in FO processes, we have found that surface ionic interactions dominate gypsum scaling on the membrane surface. A 70% flux reduction was observed on negatively charged CA and PBI membrane surfaces, due to strong attractive forces. The PBI membrane surface also showed a slightly positive charge at a low pH value of 3 and exhibited a 30% flux reduction. The atomic force microscopy (AFM) force measurements confirmed a strong repulsive force between gypsum and PBI at a pH value of 3. The newly developed PBI-POSS/PAN membrane had ridge morphology and a contact angle of 51.42° ± 14.85° after the addition of hydrophilic POSS nanoparticles and 3 min thermal treatment at 95 °C. Minimal scaling and an only 1.3% flux reduction were observed at a pH value of 3. Such a ridge structure may reduce scaling by not providing a locally flat surface to the crystallite at a pH value of 3; thus, gypsum would be easily washed away from the surface.

## 1. Introduction

Water scarcity has become one of the most challenging and imperative worldwide problems, because one third of world population is suffering from clean water shortage now, and by 2025, the number will increase to two thirds. Of all approaches to alleviate water scarcity, membrane technologies provide promising solutions by means of desalination and water reuse [1]. Other than traditional and mature membrane technologies, forward osmosis (FO) has recently drawn much attention because of several advantages, such as its unique transport mechanism, low pressure [2,3,4] and low fouling propensity [5,6,7]. However, fabrication of advanced FO membranes and the design of suitable draw solutions still require in-depth investigations in order to make FO-based water production technologies economically viable and environmentally friendly [3,8].

Water reuse by osmotic membrane bioreactors (OMBR) and desalination by FO–reverse osmosis (FO–RO) hybrid systems have been proposed and studied [6,9,10,11,12,13]. It was found that the orientation of asymmetric membranes had a significant impact on fouling tendency. Membranes oriented in the FO mode (the dense layer facing the feed solution) had much lower fouling propensity than those in the pressure retarded osmosis (PRO) mode (the dense layer facing the draw solution). Moreover, by means of osmotic backwashing [5] or intermittent tap water flushing of the membrane surface [13,14], the fouling layer in the FO mode could be effectively removed and the water flux could be easily restored. Hence, in the FO–RO hybrid system, not only was fouling on the FO membrane for seawater desalination negligible, but also fouling on the RO membrane was highly reduced [11,15]. Other factors, such as temperature and initial permeate flux, also play important roles on the degree of fouling. A high temperature was found to enhance membrane performance, but might induce severe fouling in the osmotic-driven system [16]. A high initial permeate flux of the FO membrane also led to severe fouling, because of a large permeate drag. In summary, FO processes offer the advantages of a low fouling in the FO mode and a high cleaning efficiency. Fouling in FO is more reversible than in RO, due to a lack of hydraulic pressure. The governing factors for the rate and extent of fouling are mainly determined by foulant-foulant interactions, foulant-membrane interactions and hydrodynamic interactions [14,17].

Industrial wastewater, agricultural drainage water and brackish ground water contain high levels of calcium, sulfate and carbonate ions [18,19,20]. Reuse of wastewater from the above sources by means of membrane processes encounters serious scaling issues, because these ions may reach supersaturation states and result in salt precipitation on membrane surface as foulants. Being the most common crystal phases of calcium sulfate and calcium carbonate, gypsum (CaSO_4_·2H_2_O) and calcite (CaCO_3_) are two major potential scalants during water reuse. Calcite scaling can be prohibited by lowering the pH level (e.g., pH = 5.6) of the solution, while gypsum is not as sensitive as calcite to the pH value. As a consequence, gypsum fouling is the major challenge during water purification for the above water sources [21,22].

Significant progress on FO membrane materials has been made in recent years, and a few reviews have summarized this [3,4,8]. Polybenzimidazole (PBI) received our attention, because it has superior hydrophilicity and chemical and thermal stability [23]. It can be used in harsh feed conditions without degradation. In addition, it has unique self-charged characteristics with an isoelectric point at about seven [24]. Thus, the PBI membrane provides a perfect material to investigate the complicated relationship between the membrane surface charge and the gypsum scaling. Several PBI-based FO membranes have been developed [25,26,27], and some showed a low fouling propensity [28]. Among them, hollow fiber membranes with a dual-layer configuration had the highest water flux [27]. We therefore chose PBI hollow fiber membranes with a dual-layer configuration for this study. 

It is believed that this fundamental study would provide valuable insights on gypsum fouling due to different membrane materials, surface charges and surface morphologies.

## 2. Materials and Methods

### 2.1. Membrane Materials and Module Fabrication

Three different membranes, *i.e.*, cellulose acetate (CA), polybenzimidazole (PBI)/polyethersulfone (PES) and PBI/polyacrylonitrile (PAN) with polyhedral oligomeric silsesquioxane (POSS) nanoparticles in the PBI selective layer, were used in FO tests. These membranes were fabricated using the same methods reported by Su *et al*. [29], Yang *et al*. [27] and Fu *et al*. [30], respectively. The POSS nanoparticles (AL0136) were supplied by Hybrid Plastics Inc., USA. The as-spun PBI-POSS/PAN membrane was annealed in a hot water bath at 95 °C for 3 min. All fibers were soaked in 50 wt % glycerol aqueous solutions for at least two days and air-dried at room temperature before making the membrane modules.

**Figure A1:**
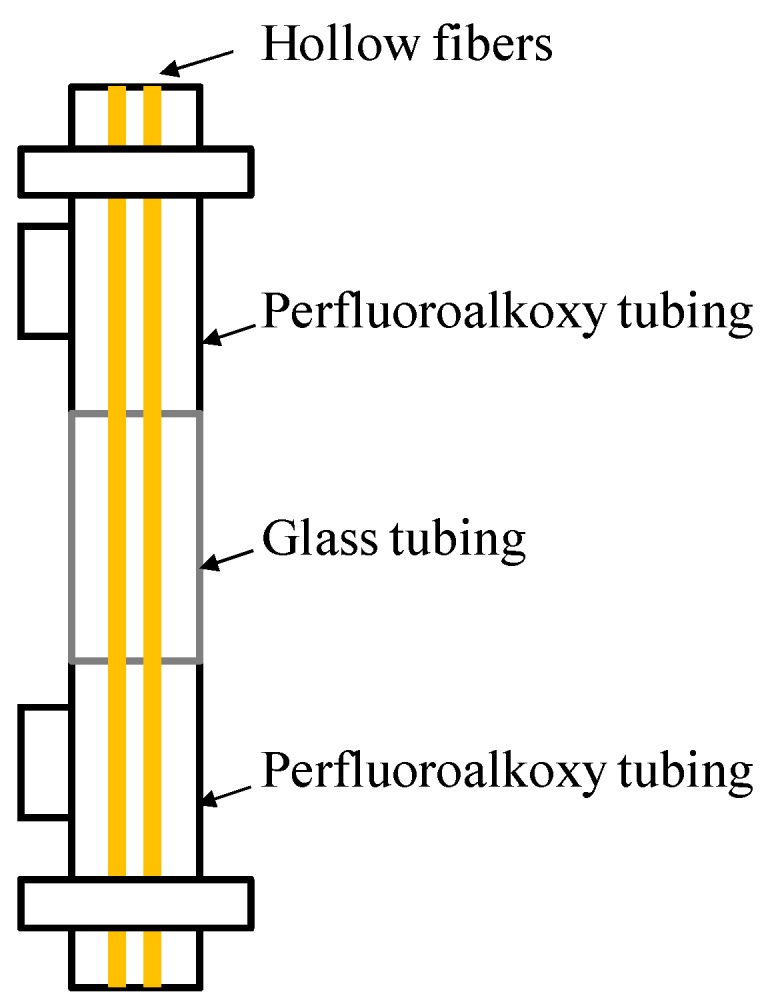
A schematic diagram of membrane modules for *in situ* observation.

Five pieces of hollow fibers with a length of 14 cm were bundled into perfluoroalkoxy (PFA) tubing with an outer diameter of 3/8 inch. The two tubing ends were sealed by an epoxy resin to assemble the membrane module for fouling studies. For *in situ* observation, a special module consisting of only two pieces of hollow fibers, as shown in Figure A1 (Appendix A), was designed for scaling experiments. The middle part of the PFA tubing was replaced by a piece of transparent glass tubing for easy and clear *in situ* observation.

### 2.2. Membrane Characterizations

#### 2.2.1. Nanofiltration Measurements

Pure water permeability (PWP) in L/m^2^ bar h and salt rejection were measured through an FO system [29] by circulating DI water and a 1000 ppm MgCl_2_ solution at the shell side of membrane modules, respectively. All nanofiltration experiments were conducted at a transmembrane pressure of 1 bar and a room temperature of 24 ± 2 °C. The permeate was collected from the lumen side and analyzed and used for the calculations of *PWP* and *R* as follows:

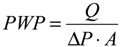
(1)

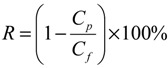
(2)
where *PWP* is in L/m^2^ bar h, *Q* is the flux in L/h, Δ*P* is the transmembrane pressure in bar, *A* is the membrane surface area in m^2^, *R* is the rejection coefficient (%) and *C_f_* and *C_p_* are solute concentrations in the feed and the permeate, respectively. The concentrations of MgCl_2_ in the feed and permeate were determined using a calibrated conductivity meter (Lab 960, Schott).

#### 2.2.2. Surface Morphology Measurements

The surface roughness of each hollow fiber membrane was measured using atomic force microscopy (AFM) (Agilent 5500 AFM). In addition to measuring the flux decline through on-line experiments, the distribution and surface coverage of gypsum particles on the membrane surface were examined using field emission scanning electron microscopy (FESEM, JOEL JSM-6700).

#### 2.2.3. Zeta Potential Measurements

The zeta potential of PBI was studied by a SurPASS electrokinetic analyzer (Anton Paar GmbH, Austria). PBI flat sheet membranes were prepared by casting the same polymer solution as for the preparation of hollow fiber membranes on a glass plate using a 100 μm gap blade. The membranes were immersed immediately into water. The zeta potential measurements followed the procedures reported by Sun *et al*. [31]. A 0.01 M NaCl solution was circulated across the PBI surface in the measuring cell of the analyzer at a maximum pressure of 500 mbar. The conductivity and pH value of electrolyte solutions were recorded to calculate the apparent zeta potential using the classic Helmholtz-Smoluchowski equation. Manual titrations by 0.1 M HCl and 0.1 M NaOH were then conducted to unravel the pH effects on the zeta potential and to determine the isoelectric point.

#### 2.2.4. Contact Angle Measurements

Contact angle θ was examined using a KSV Sigma 701 tensiometer (±0.01°, KSV Instruments Ltd.). The hollow fiber membranes were dipped into DI water, and the interfacial force was measured using a micro-balance. Through the force tensiometry method, the dynamic contact angle was then determined. Three measurements of each type of membrane were conducted, and the average value was reported.

### 2.3. Forward Osmosis Tests

FO tests were conducted through a bench-scale FO system described in [29]. The DI water or scaling solution was circulated in the shell side contacting the PBI layer (*i.e.*, the selective layer), while the draw solution MgCl_2_ was in the lumen side facing the porous support layer. The weight change of the draw solution was monitored during the experiment using a balance (AND EK-4100i). All experiments were conducted at room temperature.

### 2.4. Baseline Experiments

Baseline experiments were carried out by circulating a fresh 2 L feed solution consisting of 70 mM MgCl_2_, 40 mM Na_2_SO_4_ and 38 mM NaCl and a fresh 2 L draw solution comprised of 2 M MgCl_2_ in the shell and lumen sides, respectively. A cross-flow velocity of 23 cm/s (*Re* = 2992) was applied in the shell side, while a velocity of 51 cm/s (*Re* = 823) was maintained in the lumen side to prevent pressure build up. The CA and PBI surfaces were examined due to their distinct charge properties in acidic and basic solutions. The purpose of replacing CaCl_2_ with MgCl_2_ in the feed solution was to prevent gypsum scaling, but to keep the same ionic effect. Since the baseline experiments were conducted at pH 3 and pH 10, the effects of pH on the permeate flux will be reflected in the baseline experiments. Therefore, a comparison of pH effects between the baseline and fouling experiments show the differences, mainly due to gypsum scaling. Thus, the quantitative effects of gypsum scaling on membrane performance could be easily analyzed.

### 2.5. Gypsum Scaling Experiments

For easy comparison, the same procedures were conducted to study gypsum scaling experiments on all membrane surfaces, *i.e.*, CA, PBI, as well as PBI-POSS surfaces. The composition of scaling solutions was 70 mM CaCl_2_, 40 mM Na_2_SO_4_, and 38 mM NaCl. The corresponding saturation index of gypsum calculated by Visual MINTEQ software was 0.5. The saturation index (*SI*) is defined as follows:

*SI* = log *IAP* − log *K_s_* (3)

where *IAP* is the ion activity product and *K_s_* is the equilibrium constant. If *IAP* = *K_s_* or *SI* = 0 (−0.2 < *SI* < 0.2), the solution is saturated with gypsum. If *IAP* > *K_s_* or *SI* > 0, the solution is supersaturated, and gypsums form. If *IAP* < *K_s_* or *SI* < 0, the solution is undersaturated, and gypsums dissolve. If the *SI* value of gypsum is much greater than 0, the scaling solution has a very high tendency to form gypsum particles spontaneously. The deposition of gypsum particles on the membrane surface was studied as functions of membrane materials, charged surfaces and surface morphologies.

In each experiment, a 2 L scaling solution was circulated through the shell side of the module with a flow rate of 1 L/min, while a 2 L draw solution was conveyed through the lumen side with a flow rate of 0.1 L/min. The use of various MgCl_2_ concentrations as draw solutes is to obtain the same initial flux of 8.5 L/m^2^ h for all types of membranes. MgCl_2_ instead of NaCl was employed as the draw solute, because the former had a much smaller salt leakage than the latter. As a consequence, the possible influence from the reverse salt flux on gypsum scaling could be minimized.

The addition of NaCl in the feed solution was to mimic brine water. Moreover, the enhancement of ionic strength could lower the free calcium ion activity [19]. A low free calcium ion activity might lead to an increase in the induction time for the formation of the incipient nuclei. As a result, it gave us sufficient time to accurately monitor the scaling process. In this study, the abrupt change of water flux due to the gypsum scaling usually took place at around 200–400 min, while the duration for each fouling test was 600 min in order to cover the entire process.

To investigate the effects of the foulant-membrane interaction, the Ph value of scaling solutions was adjusted to Ph = 3.0 ± 0.1 using HCl and to Ph = 10.0 ± 0.1 using NaOH without any buffer solution. At the end of each experiment, the Ph value was re-measured, and the gypsum crystal morphology on the membrane surface was observed by microscope imaging and analyzed. The *in situ* monitoring was carried out. The membrane module made of transparent tubing was placed under an optical microscope for direct observation.

The morphologies of gypsum crystals at different Ph conditions at steady state were observed using a microscope. A few drops of freshly prepared scaling solutions at Ph 3 and Ph 10 were taken and dispersed on the petri dish to allow air drying. The gypsum crystals were formed in the petri dish when water was evaporated.

### 2.6. Physical Cleaning of Membranes

Membrane cleaning was conducted immediately after fouling experiments. It lasted for 30 min by means of air bubbling in a concentrated 0.5 M MgCl_2_ solution. The efficiency of cleaning was determined by Equation (4):

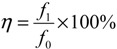
(4)
where *f*_0_ is the water flux of the fresh membrane and *f*_1_ is the water flux of the membrane after physical cleaning. The operating conditions were the same as described in Section 2.5. Subsequently, the system was washed thoroughly by DI water until the system conductivity was lower than 1 μs/cm, to ensure that all gypsum crystals were removed from the system.

### 2.7. Force Measurements at the Atomic Level

An AFM was used to measure molecular interactions between the membrane and foulants. Functionalized AFM probes were prepared by adhering one gypsum crystal onto the tipless SiN AFM cantilever using an epoxy resin using similar procedures as described by Mi and Elimelech [14]. 

The force measurement was performed in a fluid cell of the AFM. Each test used a new fiber adhered on the silicon wafer by double-sided tape. For each sample, the force between the functionalized gypsum probe and the hollow fiber was firstly measured in a saturated CaSO_4_ solution. Then, the test solution was adjusted to either an acidic or basic condition by adding a few drops of 1 M HCl or 1 M NaOH. The atomic interfacial forces between different membrane surfaces and the foulants were determined. Ten measurements of the retracting force at 10 different locations were taken and averaged.

## 3. Results and Discussion

### 3.1. Membrane Characterizations

#### 3.1.1. Membrane Structure and Morphology

**Figure 1:**
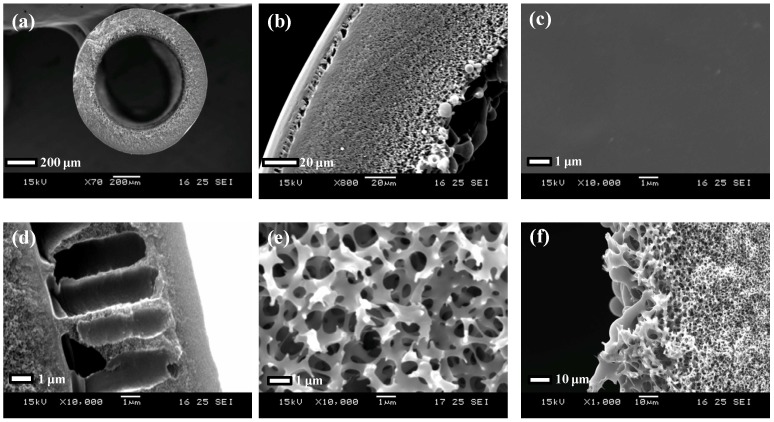
SEM images of the polybenzimidazole (PBI)/polyethersulfone (PES) membrane: (**a**) the cross section; (**b**) an enlarged cross section; (**c**) the outer PBI surface; (**d**) the outer PBI selective layer; (**e**) the middle PES transit layer; and (**f**) the inner PES support layer.

**Figure 2:**
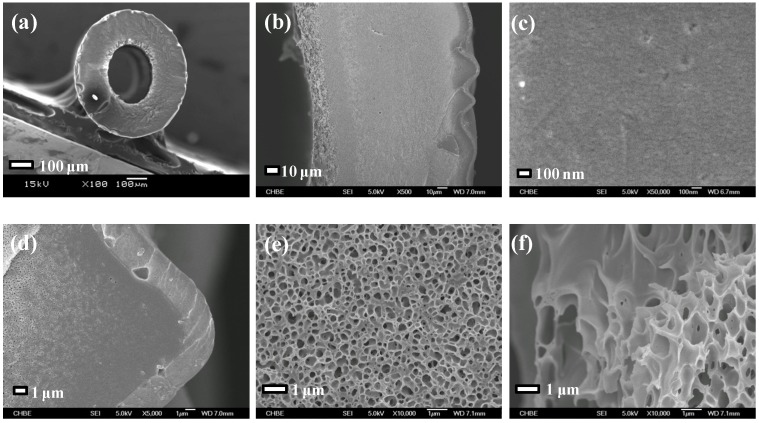
SEM images of the annealed PBI-polyhedral oligomeric silsesquioxane (POSS)/polyacrylonitrile (PAN) membrane: (**a**) the cross section; (**b**) an enlarged cross section; (**c**) the outer PBI surface; (**d**) the outer PBI selective layer; (**e**) the middle PAN transit layer; and (**f**) the inner PAN support layer.

Figure 1 and Figure 2 illustrate the SEM images of the annealed PBI/PES membrane and the annealed PBI-POSS/PAN membrane, respectively, while the SEM images of the CA membrane have been published elsewhere [29].

As shown in Table 1, the PBI-POSS/PAN membrane without annealing has the largest average wall thickness of 206 ± 1 μm, while the CA membrane has the smallest wall thickness of 110 ± 7 μm. Although the annealed PBI/PES membrane and the annealed PBI-POSS/PAN membrane have close wall thicknesses and similar macrovoid-free cross-section morphology, their average PWP values are quite different (*i.e.*, 0.56 ± 0.09 *vs*. 0.85 ± 0.11 L/m^2^ bar h). The CA membrane, as-spun and annealed PBI-POSS/PAN membranes have similar rejections to 1000 ppm MgCl_2_ (*i.e.*, 74.07% ± 3.44%, 78.0% ± 2.15%, 77.1% ± 0.91%, respectively), while the annealed PBI/PES has the highest rejection (*i.e.*, 96.33% ± 1.33%). Compared to MgCl_2_ rejections, all membranes have lower NaCl rejections, because the hydrated ionic radius of sodium ions is much smaller than magnesium ions. 

**Table 1:** Membrane dimension and basic performance characteristics.

Membrane fibers	OD (μm)	ID (μm)	Wall thickness (μm)	PWP (L/m^2^ bar h)	1000 ppm MgCl_2_ rejection (%)	1000 ppm NaCl rejection (%)	Contact angle (°)
Annealed CA	947 ± 14	726 ± 13	110 ± 7	0.89 ± 0.07	74.07 ± 3.44	56.83 ± 1.00	76.54 ± 4.41
Annealed PBI/PES	1100 ± 19	730 ± 25	185 ± 6	0.56 ± 0.09	96.33 ± 1.33	48.17 ± 0.67	63.42 ± 2.30
As-spun PBI-POSS/PAN	1038 ± 5	626 ± 4	206 ± 1	3.23 ± 0.20	78.0 ± 2.15	27.8 ± 1.96	58.50 ± 3.53
Annealed PBI-POSS/PAN	901 ± 14	523 ± 16	189 ± 11	0.85 ± 0.11	77.1 ± 0.91	46.4 ± 0.71	51.42 ± 14.85

Notes: (1) PWP (pure water permeability): DI water was circulated at the shell side of membrane modules and tested at 1 bar; (2) MgCl_2_ rejection: A 1000 ppm MgCl_2_ solution was circulated at the shell side of membrane modules and tested at 1 bar; (3) CA = cellulose acetate; OD = outside diameter; ID = inner diameter.

Table 2 compares the water fluxes (*J_f_*) and the reverse salt fluxes (*J_s_*) of these membranes under the FO mode. With a draw solution of 2 M MgCl_2_, the *J_f_* value of CA membranes is 11.9 ± 0.1 L/m^2^ h, which is the lowest due to the effect of significant internal concentration polarization (ICP). However, the CA membranes have the smallest *J_s_* value of 2.3 ± 0.3 g/m^2^ h. In contrast, PBI/PES membranes exhibit a bit higher *J_f_* of 13.6 ± 0.2 L/m^2^ h and *J_s_* of 2.7 ± 0.5 g/m^2^ h. The *J_s_*/*J_f_* ratios of both CA and PBI/PES membranes are comparable, which are 0.19 and 0.20, respectively. Without thermal annealing, the as-spun PBI-POSS/PAN membranes have a high *J_f_* of 17.6 ± 0.8 L/m^2^ h and a high *J_s_* of 27.6 ± 0.9 g/m^2^ h. After 3 min of thermal annealing in hot water at 95 °C, the *J_f_* value reduces to 12.6 ± 0.3 L/m^2^ h and the *J_s_* value reduces to 8.8 ± 0.6 g/m^2^ h. These reductions of *J_f_* and *J_s_* are mainly due to the pore size shrinkage of the PBI layer during the thermal annealing process. 

**Table 2:** Forward osmosis (FO) performance of CA, PBI/PES, as-spun PBI-POSS/PAN and annealed PBI-POSS/PAN hollow fiber membranes.

Membrane	Flux *J_f_* (L/m^2^ h)	Reverse salt flux *J_s_* (g/m^2^ h)	*J_s_*/*J_f_* ratio
CA	11.9 ± 0.1	2.3 ± 0.3	0.19
PBI/PES	13.6 ± 0.2	2.7 ± 0.5	0.20
As-spun PBI-POSS/PAN	17.6 ± 0.8	27.6 ±0 .9	1.56
Annealed PBI-POSS/PAN	12.6 ± 0.3	8.8 ± 0.6	0.69

Notes: feed solution: DI water; draw solution: 2 M MgCl_2_.

**Figure 3:**
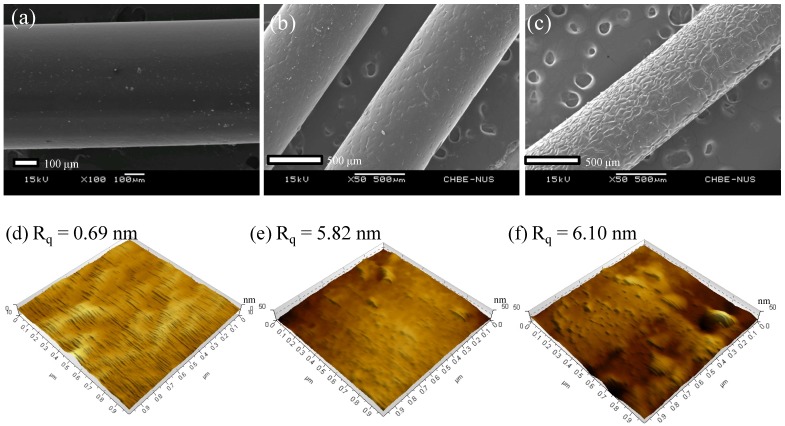
The SEM images of the outer surfaces of (**a**) the annealed PBI/PES membrane; (**b**) the as-spun PBI-POSS/PAN membrane and (**c**) the annealed PBI-POSS/PAN membrane. Only the annealed PBI-POSS/PAN membrane exhibits a micrometer-scale ridge and valley structure. The AFM images of (**d**) the CA membrane; (**e**) the PBI/PES membrane and (**f**) the annealed PBI-POSS/PAN membrane. The *z*-axis (thickness direction) scale is 10 nm for the CA membrane and 50 nm for the PBI membranes, and the scales for *x*- and *y*-axes are 1 μm for all.

Moreover, a unique micrometer-scale ridge and valley structure (Figure 3c) is observed on the annealed PBI-POSS/PAN membrane. In contrast, the annealed PBI/PES (Figure 3a) and as-spun PBI-POSS/PAN membranes (Figure 3b) do not show such a structure. The appearance of the ridge and valley structure may be a result of different degrees of shrinkage between the PBI and PAN layers.

#### 3.1.2. Surface Roughness

As shown from the AFM images in Figure 3d, the CA membrane surface is the smoothest among the three, with a root mean square roughness (*R_q_*) of 0.69 nm, which may contribute to the low fouling propensity of the CA membranes. In contrast, the annealed PBI/PES membrane and the annealed PBI-POSS/PAN membrane show similar *R_q_* of 5.82 nm in Figure 3e and 6.10 nm in Figure 3f, respectively.

#### 3.1.3. Zeta Potential of the PBI Membrane

**Figure 4:**
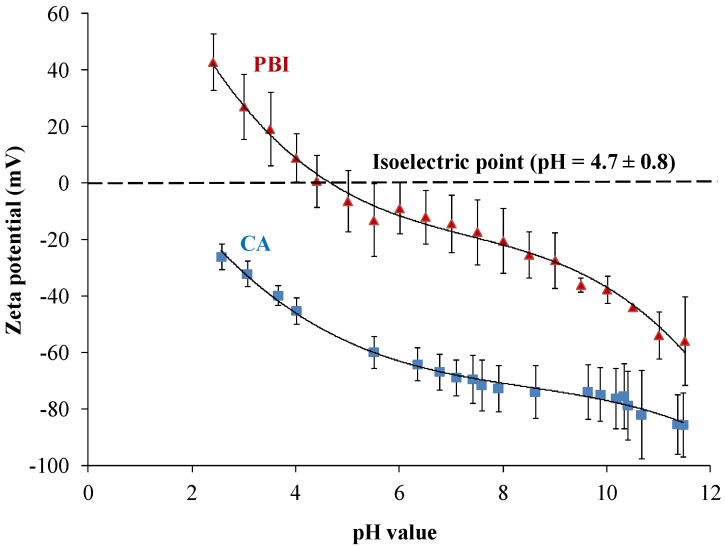
Zeta potential of the (**a**) PBI membrane and (**b**) CA membrane as a function of pH value in 0.01 M NaCl.

**Figure 5:**
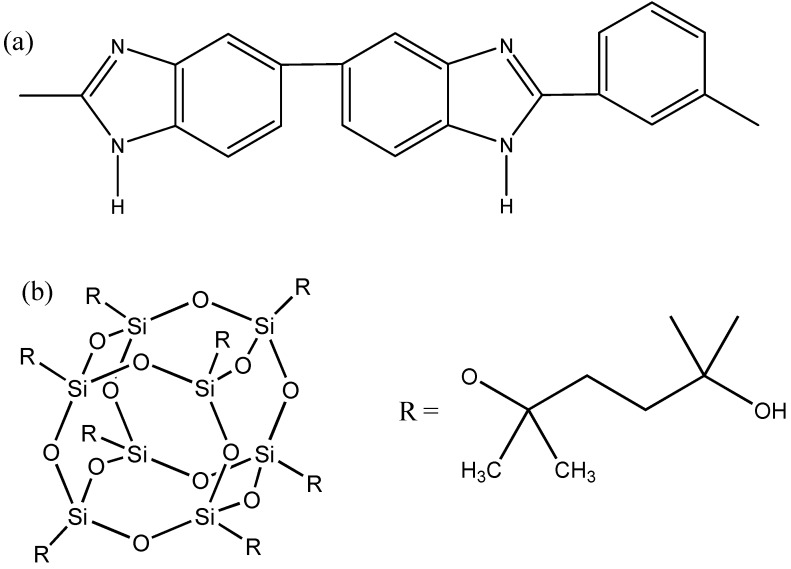
Chemical structures of (**a**) polybenzimidazole and (**b**) POSS AL0136.

As shown in Figure 4, the isoelectric point of the PBI membrane is about 4.7 ± 0.8. This means that the PBI membrane will be positively charged at a pH value of 3 and negatively charged at a pH value of 10. In contrast, the CA membrane was negatively charged from pH 2.5 to pH 11.5 (Figure 4). The amphoteric charging characteristic comes from imidazole groups of PBI (Figure 5a). The protonation and de-protonation of imidazole groups leads to the positively charged surface and negatively charged surface, respectively.

#### 3.1.4. Contact Angle

The contact angle indicates the hydrophilicity of materials. As shown in Table 1, the average contact angles of CA, annealed PBI/PES, as-spun PBI-POSS/PAN and annealed PBI-POSS/PAN membranes are 76.54° ± 4.41°, 63.42° ± 2.30°, 58.50° ± 3.53° and 51.42° ± 14.85°, respectively. These indicate that PBI has a higher hydrophilicity than CA, and the addition of POSS in PBI further increases its hydrophilicity. The latter is due to the fact that POSS AL0136 (Figure 5b) has hydroxyl and siloxyl groups, which have stronger interactions with water molecules through hydrogen bonds and van der Waals interactions [32]. It is also interesting to note that heat treatment slightly improves the hydrophilicity of the annealed PBI-POSS/PAN membrane, possibly due to the enhanced surface roughness [33,34]. Therefore, the hydrophilic PBI-POSS/PAN with heat treatment may have the lowest fouling propensity among all types of membranes.

### 3.2. Fouling and Anti-Scaling Behavior

#### 3.2.1. Anti-Scaling Characteristics in Terms of the Reduced Percentage of Flux Reduction

**Figure 6:**
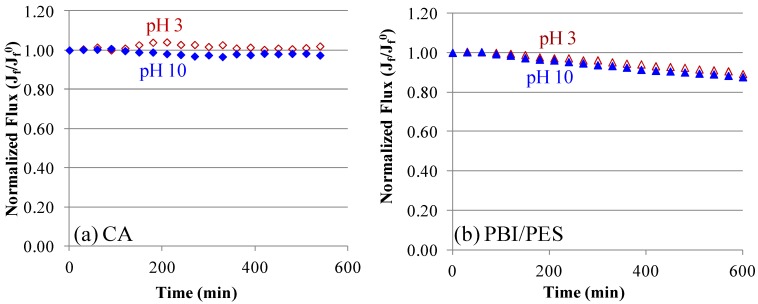
In order to compare the pH effects on CA and PBI materials, baseline experiments of CA and PBI/PES hollow fiber membranes were conducted under the FO mode. (**a**) CA membranes at pH 3 and pH 10; and (**b**) PBI/PES membranes at pH 3 and pH 10. On both CA and PBI membranes, there is no difference in flux caused by the various pH values of solutions. (The baseline solution on the shell side contained 70 mM MgCl_2_, 40 mM Na_2_SO_4_ and 38 mM NaCl with a cross-flow velocity of 23 cm/s (*Re* = 2992). The draw solution on the lumen side was 2 M MgCl_2_ at 24 ± 1 °C. The initial flux of CA is 5.5 ± 0.7 L/m^2^ h and of PBI/PES is 11.7 ± 0.7 L/m^2^ h.)

Figure 6 shows the baseline experiments on CA and PBI/PES membranes, which compare the pH effects on CA and PBI materials. Normalized fluxes, *J_f_*/*J_f_*^0^, pH 3 and pH 10 match each other closely. This is owing to the fact that the decrease of normalized fluxes is mainly due to the dilution of draw solutions, as well as the loss of salt from draw solutions. In the scaling experiments, the difference in flux between pH 3 and pH 10 will represent the pH dependence of membrane fouling.

The differences in flux decline curves result from the different induction time and different rates of scaling. Induction time determines the starting point of flux decline due to fouling, while the slope of flux decline reveals the actual rate of scaling.

**Figure 7:**
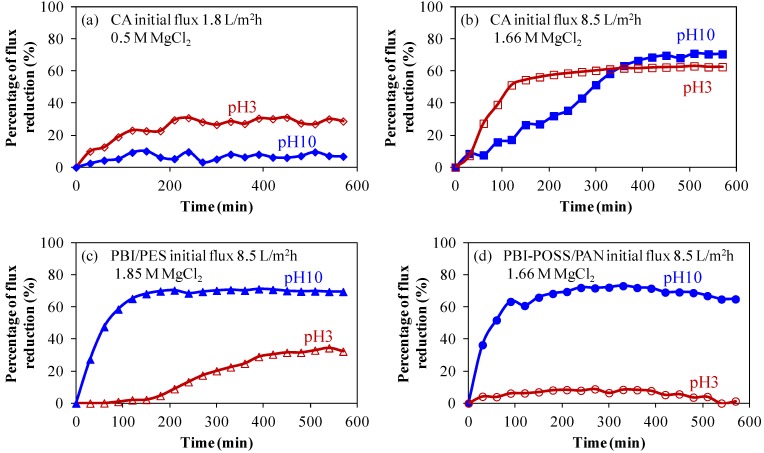
The pH effects on scaling for CA, PBI/PES and PBI-POSS/PAN membranes. CA membranes with various initial fluxes: (**a**) At a low initial flux of 1.8 L/m^2^ h, the percentage of flux reduction is limited at pH 3 (29%) and pH 10 (8%); (**b**) at a high initial flux of 8.5 L/m^2^ h, severe fouling occurs at pH 3 (63%) and pH 10 (71%). For PBI membranes with the same initial flux of 8.5 L/m^2^ h, flux reduction on (**c**) annealed PBI/PES membranes is 69% at pH 10, but only 32% at pH 3; (**d**) annealed PBI-POSS/PAN is 65% at pH 10, but only 1.3% at pH 3.

At an initial flux of 1.8 L/m^2^ h, the reference CA membrane exhibits low fouling behavior in scaling solutions at both pH 3 and pH 10, as shown in Figure 7a. Although the induction time of the formation of gypsum nuclei is longer in the pH 3 scaling solution than in the pH 10 scaling solution [19,35], the starting point of the deposition of gypsum particles on the CA membrane will not depend on the pH value of solutions significantly. In both pH 3 and pH 10 scaling solutions, the CA membrane shows a similar starting point of the flux reduction. As the initial flux increases to 8.5 L/m^2^ h (Figure 7b), the severity of fouling at pH 3 increases dramatically from a 29% flux reduction to a 69% flux reduction after 400 min fouling experiments. This observation can be explained by the concept of critical flux [36,37,38]. The increased permeation drag induced by the high permeation flux of 8.5 L/m^2^ h leads to the formation of a compact cake layer. It is worth noting that because the CA membrane is negatively charged, ranging from pH 2.5 to pH 11.5 (Figure 4), therefore, the pH effect on fouling is not as significant as the initial flux. This results in higher percentages of flux reduction at 8.5 L/m^2^ h at both pH 3 and pH 10. However, with the same initial flux of 8.5 L/m^2^ h as the CA membrane, the PBI/PES membrane exhibits low-scaling properties under a pH value lower than its isoelectric point of 4.7.

#### 3.2.2. Anti-Fouling Behavior of Positively Charged PBI Membranes

As shown in Figure 7c, the percentage of flux reduction reaches 63% for the CA membrane at a pH value of 3 (Figure 7b), but it is only 32% for the positively charged PBI/PES surface at pH 3. Clearly, ionic interactions between positively charged gypsum crystals and the PBI/PES membrane are strong enough to alter the fouling tendency. Gypsum crystals carried a positive charge, because the calcium of gypsum crystals carries two positive charges. On the other hand, the strength of ionic interactions changes direction at pH 10 for the PBI/PES membrane; thus both CA and PBI/PES membranes show high values of flux reduction.

**Figure 8:**
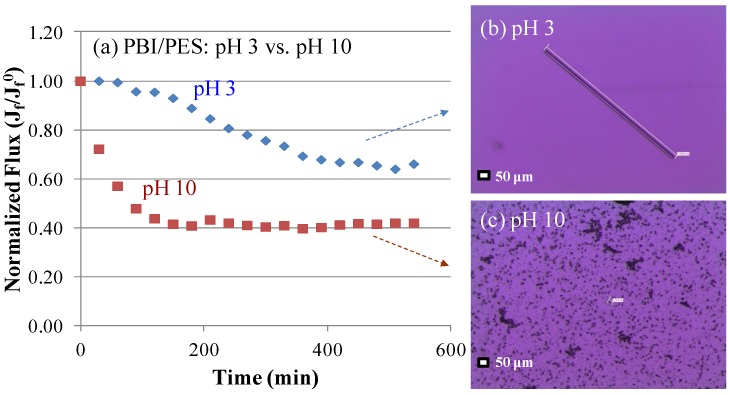
Comparison of gypsum scaling on the PBI/PES membrane at pH 3 and 10 under the FO mode. (**a**) Water flux *vs*. time at pH 3 and pH 10; (**b**) a microscopic image of gypsum crystals formed at pH 3 and (**c**) pH 10. (The scaling solution contained 70 mM CaCl_2_, 40 mM Na_2_SO_4_ and 38 mM NaCl, with a gypsum saturation index of 3.1, with a cross-flow velocity of 23 cm/s (*Re* = 2496) at 24 ± 1 °C. The draw solution was 1.85 M MgCl_2_).

As shown in Figure 7d, the annealed PBI-POSS/PAN membrane provides much lower fouling propensity than the CA and PBI/PES membranes. At pH 3, there is a 1.3% flux reduction. Moreover, SEM images show a micrometer scale structure consisting of ridge and valley (Figure 2d and Figure 3c). Because the gypsum crystals are in the scale of several micrometers (Figure 8b), this ridge structure may not provide a locally flat surface for the adhesion of crystals; thus, the newly formed gypsum would be easily washed away from the surface and result in the low fouling propensity of the PBI-POSS/PAN membrane. Formation of a smooth membrane surface at the nanoscale level, but possessing a ridge and valley structure at the micrometer level, appears to be a good strategy for the development of ultra-low fouling membranes [39,40].

Figure 8 illustrates the evolution of flux decline at different pH values for the PBI/PES membrane. It suggests that fouling progresses slowly at pH 3 in the first 200 to 450 min and then remains almost constant after 500 min. The slow process of fouling in the first 450 min may be due to the fact that PBI is positively charged at pH 3, because it has an isoelectric point of about 4.7. As a result, gypsum crystals deposit on the membrane surface slowly because of charge repulsion. Interestingly, as shown in Figure 8b, the charge repulsion may also facilitate a one-dimension growth (*i.e.*, a needle shape) of gypsum crystals in order to (1) minimize their interactions with the positively charged PBI surface and (2) to reduce the overall surface energy. Figure 8a and Figure 8c also show a distinct trend of flux decline and different crystal morphology, due to the onset of gypsum scaling at pH 10. A sharp flux decline under pH 10 is attributed to the readily adhesion of gypsum crystals onto the negatively charged PBI surface. Basically, the needle-like and oval-square shapes of gypsum crystals (Figure 8b and Figure 8c) are formed at pH 3 and pH 10 solutions, respectively.

**Figure 9:**
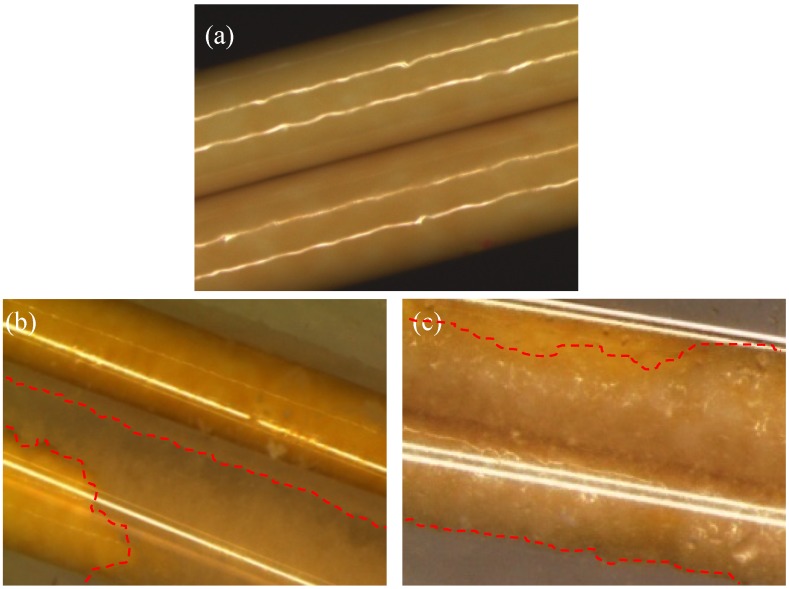
*In situ* observation of gypsum scaling on PBI/PES membranes. The yellow fiber is the PBI/PES membranes, and the white crystals are the gypsum crystals. (**a**) Clean membrane; (**b**) scaling at pH 3; and (**c**) scaling at pH 10. The percentage of surface coverage (the ratio of the membrane area scaled by gypsum over the total membrane area) on the membrane at pH 10 is higher than that at pH 3.

*In situ* observation provides direct evidence of more serious fouling behavior at a pH value of 10 (Figure 9). At both pH levels, crystals on the fiber could be observed by the naked eye as the time reached 200 min. The crystals mainly were formed at the region between fibers where there existed less shear forces from the bulk flow. Subsequently, crystals grew preferably on the existing crystals and formed clusters on the surface. At the end of the scaling experiments at pH 10, almost the whole surface of the PBI/PES membrane was covered by gypsum crystals, whereas crystals only grew in the regions between fibers at pH 3. The percentage of surface coverage by gypsum crystals (*i.e.*, the area of the membrane scaled by gypsum over the total area of the membranes) was observed significantly higher at pH 10 than at pH 3. Clearly, even though both solutions show close gypsum saturation indices, *i.e.*, 0.500 at pH 3 and 0.505 at pH 10, the attractive ionic force between gypsum crystals and the negatively charged PBI surface at pH 10 plays a determining role on fouling propensity. Furthermore, the pH value after the fouling experiment does not change much for the scaling solution with an initial pH value of 3, but decreases noticeably for the one with an initial pH value of 10. The reduced pH value for the latter case may be the result of the formation of gypsum crystals or complex; thus, the degree of alkalinity is reduced [19]. 

#### 3.2.3. Anti-Scaling Characteristics Determined by Atomic Force Measurements

**Figure 10:**
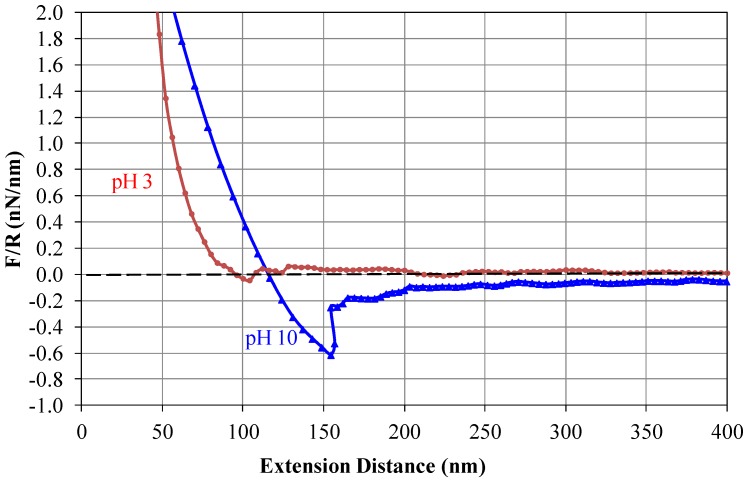
Retraction force curves on the PBI/PES membrane at pH 3 and at pH 10.

Figure 10 displays the retraction force (including van der Waals force, charge and hydrophobic/hydrophilic forces, *etc*.) between the gypsum functionalized AFM probe and the PBI membrane surface as a function of their distance. 

**Figure 11:**
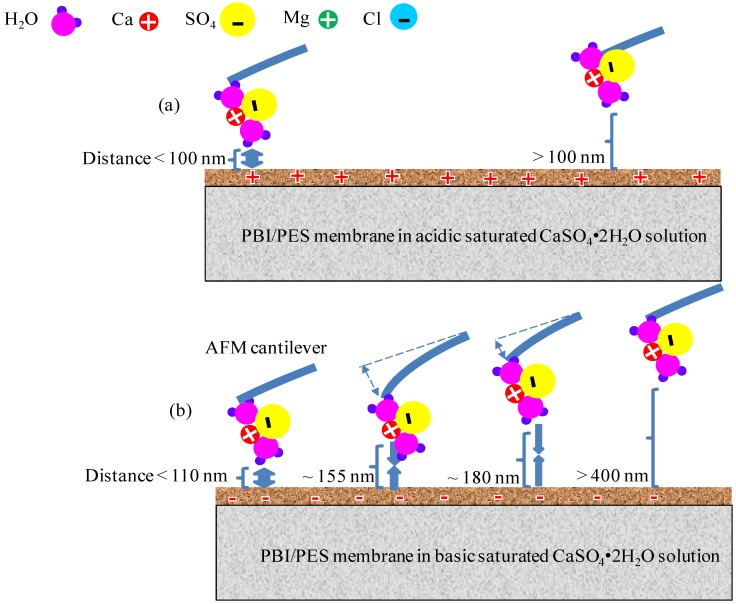
A schematic diagram of molecular interactions between the PBI/PES membrane and gypsum. (**a**) In acidic solutions, strong repulsive force exists at a distance (*i.e.*, distance between the PBI/PES membrane and gypsum) smaller than 100 nm; no attractive force can be observed; (**b**) In basic solutions, attractive force appears when the distance is larger than 110 nm (the strongest at 155 nm and the medium at 180 nm); the force disappears when distance is larger than 400 nm.

When gypsum particles and the PBI membrane surface are close to each other, the strong atomic force tends to separate them to achieve a stable energy state. As the distance increases, such atomic force also reduces. At pH 3 (Figure 11a), when the distance increases to about 100 nm, the force reduces to 0 nN, indicating that gypsum and the membrane can be readily separated if the distance between them increases further. In other words, there is no strong adhesion force between the AFM probe and the PBI membrane surface at a pH value of 3. However, at a pH value of 10 (Figure 11b), the atomic force changes from a repulsive force into an attractive force when the distance reaches about 110 nm, indicating the existence of attractive forces that resist the detachment of gypsum from the membrane. This is the major cause inducing the severe fouling at pH 10. When the distance is about 155 nm, the attracting forces reach the maximum value (*i.e.*, interaction force normalized by the radius of gypsum crystals (F/R) is 0.6 nN/nm). If the distance increases to be higher than 155 nm, the attachment breaks because of no strong attracting forces to keep them together. Usually after the break of the attachment, the force returns to zero. Thus, a decrease of force is observed at a distance of about 160 nm. It is interesting to note that when the distance is 180 nm, the attracting forces reappear (*i.e.*, F/R is 0.2 nN/nm). This means that the gypsum tends to be reattached onto the membrane surface. This is another reason for the high fouling and low cleaning efficiency at pH 10. When the distance is longer than 350 nm, the foulant-surface interaction tends to disappear. Clearly, an acidic condition induces the mutual repulsion between gypsum particles and the PBI membrane, whereas a basic condition enhances their affinity. As a result, gypsum tends to be repelled away from the PBI membrane, leading to a slower fouling and a limited flux decline at pH 3.

### 3.3. Cleaning Efficiency and Reversibility

**Figure 12:**
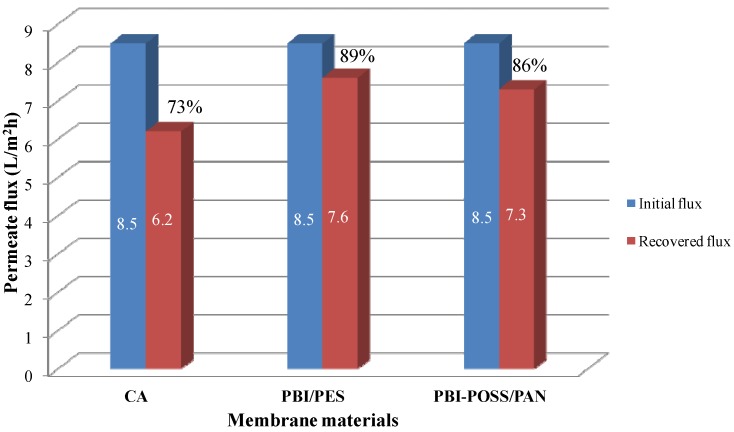
Comparison of cleaning efficiency on CA, PBI/PES and annealed PBI-POSS/PAN membranes. The initial flux was 8.5 L/m^2^ h. Blue areas are the initial permeate fluxes of fresh membranes in scaling experiments under the FO mode. Red areas are the recovered fluxes.

Figure 12 compares the initial and recovered flues of these three membranes in FO processes. After air bubbling in 0.5 M MgCl_2_ for 30 min, 73%, 89% and 86% can be recovered for CA, PBI/PES and annealed PBI-POSS/PAN membranes, respectively, with the same initial flux of 8.5 L/m^2^ h. *In situ* observation shows that turbulence induced by air bubbling vibrates the fiber and shakes away the whole piece of crystal from the fiber. Nevertheless, the salt solution during cleaning could also dissolve the crystal layer on the surface. It is observed that the crystal layer becomes thinner and thinner and finally disappears. This result can be explained by the solubility of gypsum in the presence of other salts. At 25 °C, the solubility values of gypsum are 15 mM/L in water, 19 mM/L in 50 mM NaCl and 26 mM/L in 50 mM MgCl_2_ [41,42]. 

### 3.4. Scaling Mechanisms on the Membrane Surface

**Figure 13:**
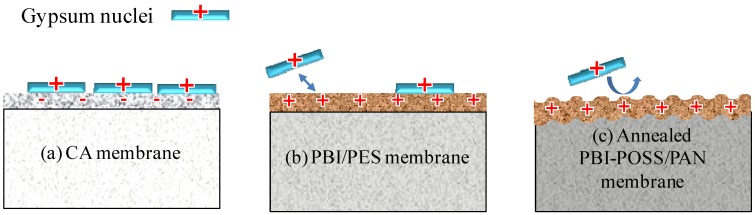
Proposed mechanisms of gypsum scaling on (**a**) the CA membrane: strong adhesion force due to the negatively charged surface; (**b**) the PBI/PES membrane: the weaker adhesion force between gypsum and positively charged surface; and (**c**) the PBI-POSS/PAN membrane: enhanced hydrophilicity of POSS and the ridge structure induced by heat treatment.

Figure 13 elucidates the fouling mechanisms on different membrane surfaces. The surface charge of CA membranes is negative in the range of pH 2.5 to pH 11. Thus, as shown in Figure 13a, the gypsum can readily attach onto the negatively charged CA membranes at a pH value of 3 and 10. Moreover, fouling on CA membranes also comes from the permeate drag. When the flux increases, the permeate drag also increases and so do the degrees of fouling at both pH 3 and 10.

The possible fouling mechanism on the PBI/PES membrane is described as follows. Because of water transport across the membrane, the scaling solution facing the membrane surface is supersaturated. Gypsum crystals are formed when the concentration of ions reach the supersaturated condition and then are attached to the membrane surface, because of the affinity between gypsum particles and the membrane surface. The onset of gypsum scaling will then impede the permeate flux. As discussed above, the positively charged membrane surface (Figure 13b) would resist the adhesion of positively charged gypsum particles, so that gypsum nuclei could not deposit onto the membrane surface easily. Therefore, fouling under pH 3 (*i.e.*, the PBI/PES membrane with a positive charge) is much slower than pH 10 (*i.e.*, the PBI/PES membrane with a negative charge).

For the annealed PBI-PAN membrane comprised of POSS, the anti-scaling properties at pH 3 can be further improved. Since the needle-like gypsum crystals formed in acidic solutions are much larger than the oval-square shapes formed in basic solutions, as shown in Figure 13c, the big crystals can be easily washed away from the membrane surface, because the ridge structure cannot provide a flat surface for adhesion. With the aid of combined effects from hydrophilic particles POSS and the ridge and valley structure induced by heat treatment, the quantity of gypsum deposited onto the membrane surface is very limited. Therefore, the extent of gypsum scaling on the PBI-POSS/PAN membranes is much less than the other two types of membranes. This observation suggests that low fouling membranes can be designed by introducing a ridge and valley structure at the micrometer scale. 

## 4. Conclusions

We have investigated gypsum scaling on the CA, annealed PBI/PES and PBI-POSS/PAN membranes with either positively or negatively charged surfaces. It was found that enhanced ionic interactions between gypsum crystals and negatively charged CA and PBI surfaces induced fast gypsum scaling. Such attractive ionic force can be alleviated by changing the PBI surface into a positively charged surface. In addition, the hydrophilic POSS particles and the micrometer-scale ridge and valley structure on the positively charged PBI membranes can further reduce gypsum scaling. This study revealed that, by manipulating the membrane surface charge, one can totally alter the affinity of crystals onto the surface. Similarly, a surface with a ridge structure can alleviate the adhesion of crystals, if the crystal size is larger the width of the valley.
